# Diabetes and the thyroid

**DOI:** 10.1186/1756-6614-8-S1-A24

**Published:** 2015-06-22

**Authors:** Magdalena Stasiak

**Affiliations:** 1 Polish Mother`s Memorial Hospital Research Institute, Lodz, Poland

## 

Thyroid disorders are more common in diabetic patients than in the general population. Abnormal thyroid function can be found in as many as 11% to 30% of patients with diabetes mellitus (DM) type 1 or 2. Thus, the Polish Society of Endocrinology and Polish Diabetes Association recommends screening for thyroid dysfunction in all patients with DM. According to these recommendations, concentrations of thyrotropin (TSH) and thyroid peroxidase antibodies (TPOAb) should be measured in every patient with newly diagnosed DM1, and in all patients with DM1 who have never undergone thyroid function tests. In exactly the same situations, patients with DM2 require TSH assessment, while TPOAb titer should be measured only if TSH reaches ≥ 2.0 mIU/L. In diabetic patients with TSH concentration ≥ 2.0 mIU/L and elevated TPOAb level, free thyroxin level should be assessed and – if normal – subsequently TSH should be monitored once a year. If the TSH concentration ≥ 2.0 mIU/L co-occurs with TPOAb titer within the reference range, TSH assessment should be repeated every other year. Patients with TSH < 2.0 mIU/L and normal TPOAb titer should undergo TSH testing every five years. Diabetic patients with a family history of chronic autoimmune thyroiditis require TSH testing once a year. If TPOAb titer is elevated in patients with DM2, the type of DM should be reassessed by measuring the level of antibodies against glutamic acid decarboxylase.

Special attention should be paid to women who are pregnant or planning pregnancy. In the preconception period, TSH concentration should be measured in every woman with DM, and in DM1 patients together with TPOAb level. When pregnancy is confirmed, assessment of TSH and TPOAb is advised at the first obstetrician appointment (before 9^th^ week of pregnancy). In all pregnant diabetic patients with a past medical history of Graves’ disease, anti-TSH receptor antibodies (TRAb) should be additionally measured at the first obstetrician appointment and repeated at the end of the second trimester (before the 22^nd^ week of pregnancy).

If carbohydrate balance is unimpaired, hyperthyroidism is rarely accompanied by hyperglycemia. Abnormal fasting or postprandial glucose levels in patients with hyperthyroidism indicate increased risk of developing diabetes. In such patients, oral glucose tolerance test (OGTT) should be performed once the euthyroidism is achieved. In diabetic patients, hyperthyroidism causes deterioration of metabolic control of DM and leads to all the systemic consequences of hyperglycemia, including increased risk of ketoacidosis. Additionally, even slight thyroid hormone excess significantly increases the risk of cardiovascular disease in patients with DM. Thus, both overt and subclinical hyperthyroidism should be treated in this group of patients. In diabetic patients with thyroid orbitopathy, the risk of optic neuropathy is increased, and intensive anti-inflammatory treatment should be introduced in every active disease except for the mild course cases. On the other hand, administering high steroid doses in patients with DM adversely influences the metabolic control of diabetes. Hence, in patients treated with oral anti-diabetic medications, periodic insulin therapy should be introduced along with steroid administration.

In diabetic patients, also the hypothyroidism is proven to be an independent risk factor for cardiovascular episodes. Therefore, treatment of subclinical hypothyroidism is strongly recommended in this condition.

Hypoglycemia or reduced insulin requirement in diabetic patients may indicate concomitant development of hypothyroidism and/ or – especially in patients with DM1 – adrenal insufficiency and polyglandular autoimmune syndrome. Unexplained hypoglycemic states always require hormonal testing to exclude these endocrine disorders.
